# Daily illumination exposure and melatonin: influence of ophthalmic dysfunction and sleep duration

**DOI:** 10.1186/1740-3391-3-13

**Published:** 2005-12-01

**Authors:** Girardin Jean-Louis, Daniel F Kripke, Jeffrey A Elliott, Ferdinand Zizi, Arthur H Wolintz, Douglas R Lazzaro

**Affiliations:** 1 Department of Psychiatry and Ophthalmology, SUNY Downstate Medical Center, New York, NY; 2 Brooklyn Research Foundation on Minority Health, KJMC, New York, NY; 3 Department of Psychiatry, Maimonides Medical Center, New York, NY; 4 Department of Psychiatry, University of California, San Diego, CA

## Abstract

**Background:**

Ocular pathology lessens light's efficacy to maintain optimal circadian entrainment. We examined whether ophthalmic dysfunction explains unique variance in melatonin excretion of older adults over and above the variance explained by daily illumination, medical, and sociodemographic factors. We also examined whether ophthalmic dysfunction influences relationships between ambient illumination and melatonin.

**Methods:**

Thirty older adults (mean age = 69 years; Blacks = 42% and Whites = 58%) of both genders participated in the study. Demographic and health data were collected at baseline. Participants underwent eye exams at SUNY Downstate Medical Center, wore an actigraph to monitor illumination and sleep, and collected urine specimens to estimate aMT6s concentrations.

**Results:**

Hierarchical regression analysis showed that illumination factors explained 29% of the variance in aMT6s mesor. The proportion of variance explained by ophthalmic factors, sleep duration, and race was 10%, 2%, and 2%, respectively. Illumination factors explained 19% of the variance in aMT6s acrophase. The proportion of variance explained by ophthalmic factors, sleep duration, and race was 11%; 17%; and 2%, respectively. Controlling for sleep duration and race reduced the correlations between illumination and melatonin, whereas controlling for ophthalmic factors did not.

**Conclusion:**

Ophthalmic exams showed that elevated intraocular pressure and large cup-to-disk ratios were independently associated with earlier melatonin timing. Lower illumination exposure also had independent associations with earlier melatonin timing. Conceivably, ophthalmic and illumination factors might have an additive effect on the timing of melatonin excretion, which in turn might predispose individuals to experience early morning awakenings.

## Introduction

Light influences numerous biological and behavioral functions [1-3]. In the laboratory, exposure to light of varying intensities, wavelengths, and durations entrains the circadian pacemaker [4], suppresses melatonin rhythms [2,3,5], and modulates pupillary reflexes [6-8]. Recent evidence suggests that these processes involve specialized signal transduction mechanisms of intrinsically photosensitive retinal ganglion cells [9]. These cells are believed to express melanopsin, the primary candidate photopigment in the synchronization of circadian rhythms [7,10-12].

Studies performed in the natural environment have shown that ambient illumination affects melatonin rhythms [13,14], rest-activity cycles [15,16], and mood [17,18]. Naturalistic studies have also demonstrated that several factors impinge on the level and timing of ambient illumination. They include age [16], gender [19], race/ethnicity [15,19], time standard [16], season [20-23], and latitude [20]. Notwithstanding the importance of these factors, the integrity of the visual and photic system remains the overriding component governing light's ability to entrain circadian rhythms.

Generally, blind patients without conscious light perception show a loss of circadian entrainment and do not experience light-induced suppression of melatonin [2,24-28]. Emerging evidence suggests, however, that a minority of blind patients maintain the capacity for photic entrainment, as demonstrated through melatonin-suppression tests [2,25]. Thus, light transmission is not necessarily abolished in all patients with no conscious light perception, particularly where no optic diseases are suspected. A recent study, investigating adolescents and young adults ages 12–20 years from the Missouri School for the Blind, found significantly greater circadian dysfunction (e.g., more daytime napping and variable timing of awakening), among patients with optic diseases relative to those without such diseases [29]. It appears that blind patients exhibiting incapacity for photic entrainment represent a unique category.

Much less is known regarding effects of age-related photic impairment on circadian rhythm functions. There are suggestions that several ophthalmic diseases could attenuate photic transmission to the circadian pacemaker. Senile miosis is one of those diseases; it is characterized by an age-related reduction in pupil diameter that could reduce retinal illumination [6,30]. Opacification and yellowing of the crystalline lens of the eye, as seen in patients with cataracts, can also substantially reduce photic transmission [31]. Loss of retinal ganglion cells, which afflicts primarily glaucoma patients, might negatively affect retinal phototransduction to the pacemaker [32,33].

It of great interest to ascertain how each of these ophthalmic diseases compromises light input to the circadian system. Judging from the available evidence, it is reasonable to hypothesize that age-associated ocular pathology lessens light's efficacy to maintain optimal circadian entrainment [34-36]. In the present study, we tested the hypothesis that ophthalmic dysfunction explains unique variance in melatonin excretion of older adults over and above that explained by daily illumination, medical, and sociodemographic factors. A parallel hypothesis examined in this study was that ophthalmic dysfunction influences the relationships between ambient illumination and endogenous melatonin rhythms.

## Methods

### Participants

Data presented in this paper were from a study investigating relations of ambient illumination to depression and melatonin excretion. Associations of daily illumination exposure with depression have been reported elsewhere [18]. The present report focuses on relationships of daily illumination and ophthalmic measures to melatonin excretion.

Respondents to study advertisements completed baseline questionnaires. They were included if they had no current eye diagnosis, their self-stated race was Black or White, were 60 years old or older, and provided informed consent under the supervision of the Institutional Review Boards at SUNY and UCSD. They were excluded if they indicated major depression or lithium use, sleep apnea, drugs that influence endogenous melatonin, a history of ocular surgery or laser treatment, or impaired cognitive or functional ability. Respondents were compensated for participating in the study.

Volunteers meeting study criteria provided demographic and health-related data, underwent eye exams, provided illumination and sleep data, and collected urine specimens. Thirty participants (mean age = 69.03 ± 6.84 years) provided complete data for the present analyses. The sample comprised Black (43%) and White (57%) Americans of both genders (women = 80% and men = 20%), with a BMI averaging 26.89 ± 6.11 kg/m^2^; 87% received at least a high school diploma and the median household income was $16,500.

### Procedures

Baseline data were acquired using the Comprehensive Assessment and Referral Evaluation (CARE), the 30-item Geriatric Depression Scale (GDS), and the Pittsburgh Sleep Quality Index (PSQI). The CARE has been widely used to assess physical health of older individuals in minority communities. It has shown good construct validity [37] as well as concurrent and predictive validity [38]. Five sub-scales were included in the present analysis: vision disorder, respiratory disease, diabetes, hypertension, and sleep disorder (Cronbach α = 0.78; 0.86; 0.82; 0.91; and 0.92, respectively).

The GDS measures depressed moods. It comprises five main factors described as: sad mood, lack of energy, positive mood, agitation, and social withdrawal. According to a study that examined depressed moods among adults (≥ 60 years old) attending primary-care clinics, the GDS had a sensitivity of 100% and a specificity of 84% in screening for major depression, using a cut-off score of 10 [39]. By contrast, the original psychometric study, which used a cut-off score of 11, found a sensitivity of 81% and a specificity of 61% for major depression (DSMIII-R) [40].

Although the PSQI is not highly specific, it is a valid measure of subjective sleep quality in clinical research. A psychometric study has shown good overall reliability coefficient for the PSQI (Cronbach α = 0.77) [41]. When investigators used a cut-off point of 5.5 in the global score, sensitivity and specificity estimates were respectively 85.7% and 86.6% for primary insomnia, 80.0% and 86.6% for major depression, 83.3% and 86.6% for generalized anxiety disorder, and 83.3% and 86.6% for schizophrenia. Nonetheless, this scale does not necessarily distinguish between conditions disturbing subjective sleep.

### Ophthalmic assessment

A trained technician performed standard examinations to assess ophthalmic disorders. These provided data on visual acuity, visual field defects (mean deviation), intraocular pressure (IOP), vertical and horizontal cup-to-disk ratios (CDR), and nerve-fiber-layer (NFL) thickness; a large CDR is an indicator of glaucoma. An ophthalmologist graded ocular photos.

Snellen best-corrected visual acuity was obtained and converted into logMAR units; higher scores denoted worse visual acuity. The SITA standard program of the Humphrey Field Analyzer was used for visual field testing to estimate ocular nerve loss [42]. Results of the Ocular Hypertension Treatment Study suggested that 97% of visual field examinations are reliable [43]. Tonometry was used to assess intraocular pressure [44,45]. The Egna-Neumarkt Glaucoma study revealed that the sensitivity and specificity of tonometry in recognizing glaucoma are 80% and 98%, respectively [44]. Fundus photography was used to examine the retina and the macula [45]. Vertical and horizontal CDR in the optic disk were derived, with higher scores indicating greater abnormality. According to the Early Treatment Diabetic Retinopathy Study, agreement rates range from 78% to 83% between retinal specialists and photographic graders [46]. Peripapillary NFL thickness, a measure of atrophy of the retinal ganglion cells, was assessed with a scanning laser polarimeter (Nerve Fiber Analyzer GDX) [47]. The GDX can detect glaucomatous eyes with a sensitivity of 71% and a specificity of 91% [48].

### Illumination and sleep assessment

Upon completion of eye exams, participants wore the Actiwatch-L (Mini Mitter Co., Inc.) for a week at home to monitor ambient illumination and sleep. The Actiwatch-L is a monitoring device worn on the wrist, which incorporates a photometer and a linear accelerometer. The photometer registers illumination that ranges from 1 to 150,000 lux. Registered lux values are averaged across each minute and stored in memory.

Illumination time-series data were imported into a computer program for least-squares cosine analyses using Action3 software. This technique is preferred because it corrects for biases due to the time of day when the recordings began and for missing data due to actigraph removal. Cosine analyses were performed on the logarithm of measured illumination. Derived circadian measures were: 1) the mesor, the fitted 24-hour average of logged illumination levels and 2) the acrophase, the timing of the peak of the fitted cosine; goodness of fit for the cosines averaged 0.65 ± 0.12. Acrophases could be linearized before performing statistical analyses, since their distribution did not cover the whole range of 360 degrees.

The accelerometer of the Actiwatch-L is sensitive to 0.01 g. and has a sampling frequency of 32 Hz; it summates and records the degree and intensity of motion on a minute-by-minute basis. Actigraphic sleep time was estimated using an automatic algorithm provided by the Actiwatch manufacturer [49]. Acceptable correlations have been found between actigraphic and polysomnographic estimates of sleep duration, but the accuracy of the algorithm has not been systematically ascertained for use among older adults. Illumination and sleep log data were used to verify time-in-bed intervals before estimating sleep and wakefulness. Sleep duration was averaged across all seven days, and this was used as a measure of habitual sleep time.

### Melatonin assessment

Urine samples were collected for approximately 24 hours near the end of the Actiwatch-L recording. Participants collected each fractional urine specimen, measured and recorded its time and total volume, and froze duplicate aliquots in two 2 cc vials. Most volunteers provided the required 10 samples spanning at least 24 hours, and most included at least one mid-sleep collection. Samples were retrieved by a staff member and sent to UCSD where they were stored at -70°C until assay of 6-sulfatoxymelatonin (aMT6s), the major urinary melatonin metabolite using 96 well ELISA kits (Buhlmann Labs, EK-M6S) purchased from ALPCO, Ltd. (Windham, NH). This is a competitive immunoassay that uses a highly specific rabbit anti-6-sulfatoxymelatonin antibody and a second antibody capture technique. Assay performance has been extensively validated by the manufacturer and results correlate well with the Arendt (Stockgrand, Ltd) RIA (r = 0.987). At the usual dilution of 1:200 the analytical sensitivity of the ELISA is 0.35 ng/ml and the functional least detectable dose (for CV < 20%) is 1.3 ng/ml. In our laboratory, control urine samples averaging 4–6 ng/ml give intra- and inter-assay CVs of 4% and 7%, respectively.

To ensure reliability of the aMT6s data, we visually analyzed excretion curves of all participants to record an overall quality score for each 24-hour profile. This evaluation was performed blind to all other information about the participants and was mainly based on the shape and completeness of the ng/h curve, but agreement between ng/h and ng/ml temporal patterns, smoothness of the baseline, and reliability of the patient log were also considered. As a circadian pattern that is clear and free of irregularities is required to estimate acrophase reliably, onset, and offset, profiles with poor quality scores were excluded. Accordingly, we selected 30 suitable profiles from a total of 59 considered. Data excluded from the final batch were not assayed due mostly to missing samples or inaccurate record keeping. Volunteers providing complete melatonin data were not significantly different in clinical presentation compared to those who did not. Of note, Blacks provided a greater number of unusable melatonin samples.

The aMT6s excretion rate for each urine sample was computed and transformed into 5-min epoch data and the resulting time series data were imported into Action3 software (Ambulatory Monitoring Inc., Ardsley, NY), where they were aligned with illumination data and further checked for accuracy. Twenty-four-hour least-squares cosine fits were computed for the full aMT6s collection (average duration, excluding missing data intervals was 24.00 h) yielding aMT6s mesors and acrophases. To estimate the duration of nocturnal aMT6s excretion, the onset and the offset of the excretion were estimated by interpolation of times at which the excretion rate (ng/h) crossed the mesor level. The time of onset of aMT6s excretion was estimated as the upward crossing and offset as the downward crossing of the mesor level; aMT6s duration was defined as the interval between onset and offset times. Goodness of fit for the cosines averaged 0.81 ± 0.11.

### Statistical analysis

All acquired data were merged into SPSS 10.0 for final analyses. These included ophthalmic, sociodemographic, medical, mood, illumination, sleep, and melatonin data. Distributions were checked for normality and were transformed where necessary using appropriate statistical techniques. Frequency and measures of central tendency were used to describe the sample. MANCOVA was used to examine race effects on ophthalmic, illumination, sleep, and melatonin measures. This procedure allowed correction for multicolinearity, if detected, and adjustment for multiple comparisons.

To examine which factors were predictive of the dependent variables: aMT6s mesor (fitted mean) and acrophase (timing), we employed two hierarchical regression models. This statistical modeling technique yields the proportion of variance in the dependent variable that can be explained by an additional set of factors, over and above that explained by the initial set. Accordingly, one can opt to use the restricted model component, providing results only for the initial set. One can also use the expanded model, which sequentially analyzes the independent contribution of additional sets. In the present analysis, the initial set comprised the mesor and the acrophase of illumination. Three other sets of factors: demographic, medical, and ophthalmic were entered in a stepwise manner. The first regression model used aMT6s mesor as the dependent variable and the illumination data plus three sets of factors as predictors. In the second model, aMT6s acrophase timing was used as the dependent variable, and the above factors were entered as in the first model.

Factors in these analyses were chosen because of their associations with the dependent measures and/or because of their hypothesized connection to melatonin. The selection process was based on preliminary results of the Pearson and Spearman correlations that were run to examine the magnitude of the correlation between each factor and the dependent variables and by examination of their collinearity. Results of these preliminary analyses revealed that race/ethnicity was the most important factor for the sociodemographic set (i.e., age, sex, race, education, and income). Of the medical set (BMI, hypertension, diabetes, mood, sleep duration, and sleep quality), sleep duration was chosen. Of the ophthalmic set (i.e., visual acuity, CDR ratios, IOP, visual fields mean deviation, and NFL thickness), IOP and horizontal CDR were selected; these two factors were chosen because they showed similar coefficients and because of their theoretical importance as indicators of glaucoma in the regression model.

To assess whether associations between illumination and melatonin were mediated by ophthalmic factors, partial correlations were used. In that analysis, the ophthalmic factors were controlled. In separate partial correlation analyses, effects of the demographic and medical factors were controlled.

## Results

Most participants (79%) were in good health. None were legally blind, but 30% were visually impaired based on standard criteria (best corrected vision worse than 20/40 and better than 20/200 in the better eye) [50]. Of the sample, 83% reported being satisfied with their sleep, although 61% indicated either difficulty initiating sleep, difficulty maintaining sleep, early morning awakening, or daytime napping. Moreover, 23% reported a respiratory condition, 60% hypertension, 77% arthritis, 43% vision problems, and 14% diabetes. Fifty-two percent reported social drinking, 15% indicated consumption of sleep aids, and 7% were current smokers.

On average, volunteers had a GDS score of 7.07 ± 3.69 and a PSQI score of 4.68 ± 2.80. Subjective and actigraphic estimates of total sleep time averaged 6.40 ± 1.04 hours and 7.55 ± 1.74 hours, respectively. Median ambient illumination was 565.68 lux. Median aMT6s excretion was 324.60 ng/h. The medians for the acrophases of illumination and aMT6s were 14.12 hours and 3.18 hours (after midnight), respectively. As seen in Table 1, race had significant effects on ophthalmic measures, indicating greater ophthalmic dysfunction for Blacks. In Table 2, we present results of race effects on illumination, melatonin, and sleep measures.

**Table 1:** Values represent adjusted mean ± standard error of ophthalmic measures. Data obtained for visual acuity were converted into logMAR units. Intraocular pressure and horizontal and vertical cup-to-disk ratios were log-transformed. For visual field mean deviation and nerve-fiber-layer thickness, a z-transformation procedure was used. Values were adjusted for effects of age and gender.

**MANCOVA: Race Effects on Ophthalmic Measures**				
**Variable**	**Black (mean ± SE)**	**White (mean ± SE)**	**F**	**p**

**Visual Acuity (logMAR)**	-0.27 ± 0.07	-0.18 ± 0.06	0.872	0.359
**Intraocular Pressure (mmHg)**	1.25 ± 0.02	1.18 ± 0.02	4.991	0.034
**Vertical Cup/Disk Ratio (mm^2^)**	-0.39 ± 0.06	-0.56 ± 0.05	6.090	0.020
**Horizontal Cup/Disk Ratio (mm^2^)**	-0.44 ± 0.05	-0.60 ± 0.04	5.060	0.033
**Visual Field Mean Deviation (dB)**	-0.52 ± 0.33	0.13 ± 0.27	2.064	0.163
**Nerve-Fiber-Layer Thickness (μm)**	-0.36 ± 0.32	0.53 ± 0.28	4.011	0.056

**Table 2:** Adjusted mean values ± standard error for illumination (lux), melatonin (aMT6s), and sleep measures. Values were adjusted for effects of age and gender.

**MANCOVA: Race Effects on Illumination, Melatonin, and Sleep**				
**Variable**	**Black (mean ± SE)**	**White (mean ± SE)**	**F**	**p**

**Light Mesor [log]**	1.03 ± 0.10	1.38 ± 0.08	6.033	0.022
**Light Phase [hr]**	13.57 ± 0.34	14.54 ± 0.27	4.306	0.049
**aMT6s Mesor [log]**	2.67 ± 0.14	2.31 ± 0.11	3.311	0.082
**aMT6s Phase [hr, after midnight]**	2.35 ± 0.37	3.45 ± 0.29	4.745	0.040
**Phase Angle Between aMT6s and Sleep Timing [hr]**	2.55 ± 0.43	2.25 ± 0.29	0.305	0.586
**Mid-Sleep [hr]**	4.76 ± 0.34	5.51 ± 0.27	2.580	0.122
**Sleep Duration [hr]**	5.75 ± 0.28	6.57 ± 0.21	4.800	0.039

Analysis indicated that the mesor and the acrophase of aMT6s were both associated with the sociodemographic, medical, and ophthalmic factors. The multiple correlation (r^2^) of aMT6s mesor to these factors added individually was: [r^2 ^= 0.24; r^2 ^= 0.23; r^2 ^= 0.15, respectively]; for aMT6s acrophase, it was: [r^2 ^= 0.15; r^2 ^= 0.21; r^2 ^= 0.28, respectively]. However, in the interest of developing parsimonious regression models and because our sample was too small for a detailed analysis of the overlapping effects of all of the factors on the dependent variables, we selected representative factors from each set of factors. Accordingly, besides the mesor and acrophase of illumination only race, sleep duration, CDR and IOP were entered into the hierarchical regression models as predictors. With a sample size of 30 and an alpha value set at 0.05, it was determined a priori that the study would have power of 0.85 to construct a reliable model with six predictors, accounting for 41% of the variance in the dependent variable.

Results of the first hierarchical regression analysis showed that illumination factors explained 29% of the variance in aMT6s mesor; illumination acrophase was the main contributor, indicating that individuals showing later timing had lower aMT6s mesors. Sequential addition of the other factors (i.e., CDR and IOP, entered as a set, sleep duration, and race) showed that the proportion of variance explained by each was 10%, 2%, and 2%, respectively. Overall, the expanded model accounted for 43% of the variance in aMT6s mesor [F = 3.47, p < 0.05]. The adjusted stepwise correlations of each of the factors to aMT6s mesor were: illumination mesor [r_p _= -0.08], illumination acrophase [r_p _= -0.49], race [r_p _= -0.08], sleep duration [r_p _= -0.25], IOP [r_p _= 0.31], and CDR [r_p _= -0.25]. For each of these correlations, effects of the other five factors were simultaneously adjusted.

In the second hierarchical regression analysis, where aMT6s acrophase was the dependent variable, the illumination factors explained 19% of the variance; individuals receiving greater daily illumination level and showing later illumination timing were likely to show later aMT6s timing. The proportion of variance explained by the factors: CDR and IOP (entered as a set), sleep duration, and race was 11%; 17%; and 2%, respectively. Altogether, the expanded model accounted for 49% of the variance in aMT6s acrophase [F = 2.64, p < 0.05]. The adjusted stepwise correlations of each of the factors to aMT6s acrophase were: illumination mesor [r_p _= 0.41], illumination acrophase [r_p _= 0.09], race [r_p _= -0.03], sleep duration [r_p _= 0.48], IOP [r_p _= -0.29], and CDR [r_p _= -0.32].

In Table 3, we present results of the partial correlation analyses, examining associations of illumination factors with melatonin measures. Consistent with regression results, later timing of illumination was significantly associated with lower aMT6s mesor. Controlling for sleep duration and race somewhat reduced this association, whereas controlling for IOP and CDR affected them little. Trends suggested that greater illumination was associated with later aMT6s timing.

**Table 3:** Values represent correlation coefficients (Coef.) for associations of ambient illumination with melatonin measures from three separate analyses. First, Pearson correlations were run with no control for the covariates. Second, partial correlations were run with control for sleep duration and race. Third, partial correlations were run with control for intraocular pressure (IOP) and cup-to-disk ratio (CDR).

**Relationships Between Illumination and Melatonin**					
		**aMT6s Mesor**		**aMT6s Phase**	

	**Variable**	**Coef.**	**p**	**Coef.**	**p**

*No control [r]*	**Light Mesor**	0.07	0.73	0.31	0.09
	**Light Phase**	-0.50	0.01	0.18	0.35

*Control for sleep and race [r_p_]*	**Light Mesor**	0.19	0.35	0.28	0.16
	**Light Phase**	-0.43	0.03	0.03	0.88

*Control for IOP and CDR [r_p_]*	**Light Mesor**	0.07	0.71	0.22	0.25
	**Light Phase**	-0.49	0.01	0.08	0.61

## Discussion

The data show that ophthalmic dysfunction was associated with the endogenous melatonin rhythms of community-residing older adults. Ophthalmic factors explained a significant proportion of the variance in 24-hr 6-sulphatoxymelatonin excretion (mesor) and timing (acrophase), over and above the variance explained by daily illumination, sleep duration, and race. Although most of the volunteers were in good health, ophthalmic exams showed significant evidence of photic impairment anchored by elevated intraocular pressure and large cup-to-disk ratios, which were independently associated with earlier melatonin timing. We observed that lower illumination levels also had independent associations with earlier melatonin timing. Conceivably, ophthalmic and illumination factors might have an additive effect on the timing of melatonin excretion, which in turn might predispose individuals to experience early morning awakenings.

As greater intraocular pressure and cup-to-disk ratio may be indicative of optic nerve loss, a common finding among glaucoma patients [32], their effects on melatonin rhythms might be mediated by a defect in retinohypothalamic stimulation. Unfortunately, this study did not offer direct support for this hypothesis. Ophthalmic dysfunction does not seem to have a mediating effect on the relationships between ambient illumination and melatonin rhythms, as these relationships remained virtually unchanged when we controlled for differences in ophthalmic factors. Hence, abnormalities in both IOP and CDR may have had a direct effect on the timing of melatonin excretion of White and Black participants. However, associations of IOP and CDR with the amount of melatonin excretion were mixed, with greater IOP predicting greater excretion while greater CDR predicted lower excretion rates, which was in the expected direction. This discrepancy merits further examination, but we might consider that previous studies of melatonin rhythms in uncontrolled environments have shown that the acrophase, rather than the mesor of melatonin excretion, strongly correlated to ambient illumination [13], depression scores [51], activity rhythms [52], napping behavior [53], and duration and timing of sleep [52,54].

Habitual illumination pattern was the best predictor of aMT6s rhythms of all the factors in the regression models. Both brighter and later illumination exposure correlated to later aMT6s timing, although illumination level was a better predictor in the regression model. We noted that the timing of illumination exposure, rather than its mesor, correlated significantly to the mesor of aMT6s. It might be that a later illumination acrophase reflects less illumination exposure in the morning before the endogenously timed offset of melatonin secretion. Therefore, a later illumination acrophase might be associated with less morning light suppression of melatonin and, in turn, a delayed acrophase of aMT6s excretion.

The timing of daily illumination might be a better index of the amount of aMT6s excretion, irrespective of individuals' sociodemographic and medical characteristics. Evidently, this must be balanced against the observation that the timing of melatonin excretion can be influenced by age-related weakening of the circadian pacemaker as well as by individual preferences in the timing of outdoor daylight activities [55-57]. Other factors influencing melatonin excretion in the natural setting include day length, age, duration and timing of sleep, and usage of certain medications [13,16,19,23,58]. Our analysis considered the relative contribution of all these factors, except for day length (season), but scheduling of the recordings was balanced across seasons throughout the study period.

Sleep duration is another factor that played an important role in the analyses. That sleep duration correlated with both the mesor and the acrophase of aMT6s is consistent with previous findings [59-61]. We would have expected that shorter sleep duration would correlate with reduced aMT6s excretion, as predicted by data suggesting a longer experience of nocturnal darkness (as might be associated with a longer sleep duration) results in a longer duration of melatonin excretion [62]. The inverse correlation found in our study may have been influenced by the finding that Blacks slept less than did Whites while showing greater mesors of aMT6s excretion. It is noteworthy that in our preliminary analyses aMT6s measures had stronger correlations to sleep duration than to a history of hypertension, diabetes, or respiratory disease, BMI, mood or sleep quality. Possibly, sleep duration is a proxy for these measures, as it correlates to each, albeit to varying degrees.

Of all the sociodemographic factors we analyzed, race was the strongest correlate of aMT6s measures. This is consistent with results of the analysis of covariance reported in Table 2. Independent of individuals' age and gender, race had significant effects on the melatonin measures. Similarly, race had significant effects on the ophthalmic, illumination, and sleep variables. These findings evidence that race is an important factor when analyzing sleep and circadian rhythm measures. Notwithstanding, it is less robust than the illumination, sleep, and ophthalmic factors in explaining the variance in aMT6s measures. One explanation for the reduced significance of race in the regression models relates to the shared variance in aMT6s measures explained by both race and these other factors.

Consistent with previous epidemiological and clinical data, individuals of the Black race showed worse scores on ophthalmic exams [63,64]. A thinner nerve fiber layer, an elevated intraocular pressure, and greater cup-to-disk ratios, as observed among Blacks, are three important indicators of optic nerve loss in glaucoma. One implication of these findings is that since glaucoma is more common among Blacks [65,66], they may be at increased risks of developing circadian abnormalities through reduction of photic transduction to the circadian pacemaker.

Since we used a relatively small sample size, we could not assess the overlapping effects of all the independent factors on melatonin rhythms. It was evident that daily illumination, ophthalmic factors, sleep duration, and race each had independent associations with both the mesors and acrophases of melatonin excretion. Although our regression models approximated predictions of the power analysis, they warrant replication in a larger sample. Efforts should be made to provide detailed instructions in gathering melatonin samples among minority groups. The observation that Blacks had lower illumination exposure, greater ophthalmic dysfunction, and higher aMT6s levels merits further empirical study, as these characteristics are suggestive of depressed moods [18,51,58,67].

## Competing interests

The author(s) declare that they have no competing interests.

## Authors' contributions

**GJL **supervised volunteer recruitment, data collection, data analysis, and drafting of the manuscript.

**DFK **helped design the study and assisted in data analysis and drafting of the manuscript.

**JAE **performed aMT6s assays and assisted in the drafting of the manuscript.

**WAH **participated in the analysis and interpretation of the ophthalmic data; he also assisted in the drafting of the manuscript.

**LDR **helped with the interpretation of the ophthalmic data and with the drafting of the manuscript.

All authors read and approved the final manuscript.
